# A Novel SPAST/SPG4 Splice-Site Variant in a Family with Dominant Hereditary Spastic Paraplegia

**DOI:** 10.1155/2020/7219514

**Published:** 2020-08-29

**Authors:** Nathaniel M. Robbins, Jillian R. Ozmore, Thomas L. Winder, Pedro Gonzalez-Alegre, Tanya M. Bardakjian

**Affiliations:** ^1^Department of Neurology, Geisel Dartmouth School of Medicine, Hanover, USA; ^2^Department of Medical Genetics, Dartmouth-Hitchcock Medical Center, Lebanon, USA; ^3^Invitae Corporation, San Francisco, USA; ^4^Department of Neurology, The University of Pennsylvania, Philadelphia, USA

## Abstract

Some causes of spastic paraplegia are treatable and many are not. Diagnostic work-up to determine the etiology can be costly and invasive. Here we report the case of a man with slowly progressive spastic paraparesis. Using a multigene next-generation sequencing (NGS) panel, we identified a novel variant in the consensus splice site of the SPAST gene (exon 13, c.1536G>A, heterozygous), affecting codon 512 of the SPAST mRNA. The observed variant segregated with the disease in four tested family members. In this case, genetic confirmation obviated the need for additional testing such as MRI and lumbar puncture and helped the patient and his family understand his condition and prognosis. We conclude with a brief discussion of the SPG4/SPAST gene and the role of multigene panels in the diagnosis and management of hereditary spastic paraplegia.

## 1. Case Presentation

A 46-year-old man presented with insidiously progressive gait and balance difficulties over fifteen years. His legs felt stiff. Gait was slow. He could trip from catching his toes on the ground. The lateral part of his shoes wore away asymmetrically. He endorsed urinary frequency but normal bowel function. He had occasional cramps but no significant pain. He had mild memory complaints but continued to work, drive, and do all other independent activities of daily living. He took no medications; reported no trauma; and denied substance use.

**Figure 1:**
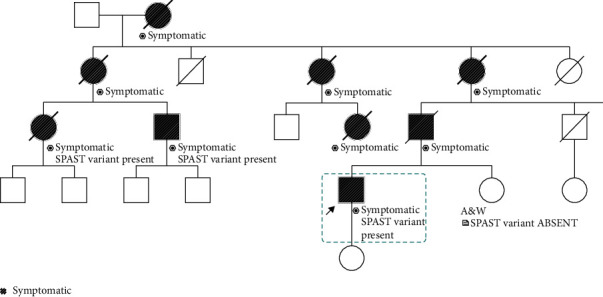
Pedigree of the family.

The proband's father developed symptoms around age 45 and cognitive problems (no additional details available) before he died at 75. A paternal cousin developed gait problems in her 30 s. She later developed urinary and bowel dysfunction as well as anosmia, ageusia, and sensorineural hearing loss. Cognition remained normal.  Figure 1 details the family history.

Neurologic examination revealed a spastic paraparesis, with symmetrically brisk (3 out of 4) patellar and ankles reflexes, and bilateral extensor responses with plantar stimulation. Ankle eversion was slightly weak (Medical Research Council 5-/5) but other distal (i.e., toe and ankle extension, foot inversion) and proximal muscles were strong. He could stand on his toes but not his heels. Vibration sensation was slightly reduced distally, but pinprick and light touch were normal. Gait was scissoring. He scored 25 of 30 on a Montreal Cognitive Assessment, losing three points for delayed recall, and one point each for clock draw and tapping “A's.” Mental status, cranial nerve, and arm examination were otherwise normal. He was clinically diagnosed with a thoracic myelopathy, presumed to be hereditary. Vitamin B12 was normal.

A twelve-gene panel looking for autosomal dominant hereditary spastic paraparesis (HSP) was sent to Invitae (San Francisco, CA—see below and Appendix for genetic testing methods). NGS identified a variant of uncertain significance (VUS) in the SPAST gene (exon 13, c.1536G>A, heterozygous), affecting codon 512 of the SPAST mRNA (NM_014946.3). This variant was not present in any standard population or clinical databases (see Appendix for databases queried, or the following web page: https://www.invitae.com/static/data/WhitePaper_Variant-Classification-Method.pdf). This variant fell at the last nucleotide of exon 13, which is part of the consensus splice site. A novel pathogenic variant was suspected since nucleotide substitutions within the consensus splice site are a relatively common cause of aberrant splicing [1] and since a different variant affecting this nucleotide (c.1536G>C) was previously reported as probably pathogenic using RT-PCR for mRNA analysis and familial segregation analysis [2]. In silico splice models were used to analyze the c.1536G>A variant (see Appendix for methods), and all models suggest this variant interferes with normal splicing, though this has not been confirmed by published transcriptional studies. Saliva from the patient's sister and father's affected cousin was then tested. Testing demonstrated the identical variant in the affected cousin, but not the unaffected sister (see pedigree—Figure 1), supporting the hypothesis that this novel variant is pathogenic, though functional expression studies were not done.

## 2. Genetic Testing Methods

The following 13 genes were analyzed for sequence changes and exonic deletions/duplications using NGS: *ALDH18A1, ATL1, BSCL2, HSPD1, KIF1A, KIF5A, NIPA1, REEP1, REEP2, RTN2, SPAST, VAMP1*, and *WASHC5*. As previously described [3–5], genomic DNA obtained from the proband's blood and family members' saliva was subjected to target enrichment using hybridization capture with a custom bait pool and sequenced using Illumina sequencing chemistry. A validated bioinformatics pipeline incorporating community standard and custom algorithms was used to identify sequence changes and exonic deletions/duplications simultaneously. Clinically significant observations were confirmed by orthogonal technologies, except individually validated variants and variants previously confirmed in a first-degree relative. Depending on the variant type, confirmation technologies may include any of the following: Sanger sequencing, Pacific Biosciences SMRT sequencing, MLPA, MLPA-seq, and Array CGH. Variants were evaluated and classified using a refinement of the five-tier system for grading evidence for pathogenicity by American College of Medical Genetics and Genomics (ACMG) [6, 7]. Appendix illustrates more detailed methods.

## 3. Discussion

A mutated SPAST gene, which encodes the microtubule-severing protein spastin, is the most common cause of HSP, accounting for 15–40% of all HSP cases [8, 9]. Autosomal dominant HSP-SPG4 is usually “uncomplicated,” causing lower limb spasticity only without shortening lifespan. However, cognitive impairment has been described, and up to 50% of patients can have autonomic symptoms [8–11]. The mechanisms by which a mutated SPAST causes dysfunction are complex and remain incompletely understood [8].

Known pathogenic variants in SPAST/SPG4 include missense, nonsense, deletions, insertions, and splice-site variants [2, 8, 9]. Age of onset ranges from infancy through senescence, and functional impairment is highly variable [2, 8, 9, 11]. Penetrance is incomplete though typically symptomatic and age-dependent, with 50% and 80% showing symptoms at age 27 and 50, respectively [8].

Insidious spastic paraparesis with few sensory symptoms and a positive family history strongly suggests HSP. However, in the absence of genetic confirmation, HSP is a diagnosis of exclusion, which can lead to expensive and superfluous MRIs, lumbar punctures, and blood tests looking for metabolic, infectious, and inflammatory causes of myelopathy. Genetic confirmation can curtail this misdirected testing and help patients to obtain psychological closure, better understand prognosis, and gain entry into clinical trials and support groups that require a diagnosis. Since more than 80 genes and many more variants have thus far been associated with HSP, NGS panels have become essential for diagnosis and should be considered first-line in the right clinical scenario [12]. Cost of NGS varies widely based on insurance coverage, but can be as little as 250 USD: far less than MRI.

Here NGS and family comparison testing identified a novel variant in SPAST associated with classic HSP-SPG4, thereby expanding the known genetic spectrum of this heterogeneous disease. A genetically confirmed diagnosis obviated the need for additional testing.

## Appendix

### Genetic testing methods used by Invitae

Clinical genetic testing for autosomal dominant hereditary spastic paraparesis was performed at Invitae (San Francisco, California). The following 13 genes were analyzed for sequence changes and exonic deletions/duplications using next-generation sequencing: ALDH18A1, ATL1, BSCL2, HSPD1, KIF1A, KIF5A, NIPA1, REEP1, REEP2, RTN2, SPAST, VAMP1, and WASHC5. As previously described [3–5], genomic DNA obtained from the proband's blood and family members' saliva was subjected to target enrichment using hybridization capture with a custom bait pool and sequenced using Illumina sequencing chemistry. A validated bioinformatics pipeline incorporating community standard and custom algorithms was used to identify sequence changes and exonic deletions/duplications simultaneously. Clinically significant observations were confirmed by orthogonal technologies, except individually validated variants and variants previously confirmed in a first-degree relative. Depending on the variant type, confirmation technologies may include any of the following: Sanger sequencing, Pacific Biosciences SMRT sequencing, MLPA, MLPA-seq, and Array CGH. Variants were evaluated and classified using a refinement of the five-tier system for grading evidence for pathogenicity by American College of Medical Genetics and Genomics (ACMG) [6, 7]. In silico splicing programs used are as follows: GeneSplicer, NNSPLICE, MaxEntScan, and SpliceSiteFinder-like.
